# Prolonged Wnt3a exposure tolerizes macrophages to inflammatory stimuli

**DOI:** 10.3389/fimmu.2026.1752131

**Published:** 2026-03-12

**Authors:** Megan L. Tigue, Rincon Jagarlamudi, Channing Chi, Diana Diaz, Hua-Chang Chen, Quanhu Sheng, Sheau-Chiann Chen, Anna Schwarzkopf, Jeremy A. Goettel, Heather H. Pua, Ethan Lee, Vivian L. Weiss

**Affiliations:** 1 Department of Pathology, Microbiology, and Immunology, Vanderbilt University Medical Center, Nashville, TN, United States; 2 Program in Cancer Biology, Vanderbilt University School of Medicine, Nashville, TN, United States; 3 Department of Biostatistics, Vanderbilt University Medical Center, Nashville, TN, United States; 4 Department of Cell and Developmental Biology, Vanderbilt University, Nashville, TN, United States; 5 Department of Medicine, Division of Gastroenterology, Hepatology and Nutrition, Vanderbilt University Medical Center, Nashville, TN, United States; 6 Vanderbilt Institute of Infection, Inflammation and Immunology, Vanderbilt University Medical Center, Nashville, TN, United States; 7 Center for Mucosal Inflammation and Cancer, Vanderbilt University Medical Center, Nashville, TN, United States

**Keywords:** macrophages, plasticity, regulation, tolerance, Wnt signaling, Wnt3a

## Abstract

**Introduction:**

Macrophages are highly plastic innate immune cells that have a broad range of phenotypic and functional roles in the body. The Wnt/β-catenin signaling pathway is known to play important roles in regulating the immune system, but the literature contains contradictory evidence for how Wnt impacts macrophages. Given the plasticity of macrophages, as well as the growing interest in utilizing Wnt inhibitors therapeutically, there is a need to better understand how Wnt signaling affects macrophage phenotype and function.

**Methods:**

We treated murine bone marrow derived macrophages with Wnt3a, LPS/IFN-γ, or IL-4 and measured gene/protein expression with bulk RNA sequencing, RT-qPCR, flow cytometry, and immunofluorescence to assess macrophage phenotype.

**Results:**

RNA sequencing of macrophages treated continually for 5 days with Wnt3a demonstrated upregulation in genes associated with chemotaxis, cytokine activity, and both pro- and anti-inflammatory phenotypes. A time-course of Wnt3a treatment revealed acute upregulation of the inflammatory cytokines *Il6*, *Tnf*, and *Il12b*. Later timepoints showed upregulation of regulatory markers, such as *Il10*. Finally, re-treating with classic inflammatory cytokines revealed a Wnt-induced tolerant phenotype.

**Discussion:**

In this study, we expanded upon past work to show that acute stimulation by Wnt3a induces inflammatory activation of macrophages in a time-dependent manner. Chronic stimulation with Wnt3a, as may be expected in a Wnt-ligand rich tissue microenvironment, caused macrophages to become tolerant to additional inflammatory stimuli and to upregulate markers of an anti-inflammatory phenotype. This study highlights the importance of considering time-dependent plasticity and regulatory feedback mechanisms in understanding macrophage phenotypes.

## 1 Introduction

Macrophages are highly plastic, innate immune cells with numerous roles in the body. Their phenotypes and functions change based on external signals from the tissue microenvironment. One way that macrophage activation can be represented is as a continuum extending between two extreme states: pro-inflammatory (M1-like) to anti-inflammatory (M2-like) (1). “Classically-activated” macrophages acquire a pro-inflammatory phenotype, secreting cytokines like TNF-α and IL-6. They play major roles in local infection control by phagocytosing bacteria and cellular debris (2, 3). *In vitro*, lipopolysaccharide (LPS) and IFN-γ are used to induce pro-inflammatory polarization of macrophages (1, 4). On the opposite end of the macrophage polarization spectrum, “alternatively-activated” macrophages, stimulated *in vitro* by IL-4 treatment, are considered to be anti-inflammatory and tissue-repairing (1, 5). In the setting of cancer, macrophages that more closely resemble alternatively-activated macrophages are considered pro-tumorigenic because they limit immune system control of tumor growth (3, 6).

Numerous signals from a tissue microenvironment work in concert to change the phenotype of macrophages. One pathway that plays crucial roles in embryonic development, tissue homeostasis, and many disease states is the Wnt/β-catenin signaling (henceforth Wnt signaling) pathway. Wnt signaling plays important roles in regulating inflammatory responses in disease, including promoting anti-inflammatory tumor microenvironments (7, 8). At the level of adaptive immunity, Wnt signaling supports CD8^+^ T cell memory formation and suppresses the differentiation of effector T cells (9, 10). Wnt signaling also mediates tolerogenic processes in dendritic cells, another important player in the innate immune system (8, 11–13). Most studies focused on the effects of Wnt signaling on macrophage polarization suggest that Wnt has a dominant role in promoting anti-inflammatory, M2-like phenotypes (14–16). Other *in vivo* data also suggest a correlation between anti-inflammatory macrophages and upregulated Wnt signaling in settings such as hepatic carcinoma and kidney fibrosis (14, 15). However, there are a few studies that suggest that Wnt signaling can promote the formation of pro-inflammatory macrophage phenotypes (17, 18). Resolving these seemingly contradictory findings would allow for improved targeting of both Wnt signaling and macrophages in disease.

In this study, we sought to validate and clarify previous findings suggesting a role for Wnt activation in macrophage polarization. We discovered that treating murine bone-marrow derived macrophages (BMDMs) with Wnt3a, a prototypical Wnt ligand and potent activator of Wnt/β-catenin signaling (19), generated a large, acute inflammatory response. Dynamic changes in gene expression were similar in BMDMs treated with LPS and IFN-γ. To date, most literature has suggested that Wnt signaling promotes M2-like polarization of macrophages. However, our results demonstrate that Wnt3a treatment supports a phenotype that more closely resembles classically activated, M1-like macrophages, which initially produce inflammatory cytokines but express genes associated with feedback inhibition and a tolerant state over time.

## 2 Methods

### 2.1 Cell culture and conditioned media preparation

L929 murine fibroblast cells (ATCC #CCL-1) were cultured in RPMI + 10% fetal bovine serum (FBS) supplemented with 1% penicillin-streptomycin (cRPMI). To produce L929 conditioned media (CM), 4.7 x 10^5^ cells were plated in a T175cm flask in 55mL cRPMI. After 7 days, the media was collected, filtered through a 0.22μm filter, and stored in aliquots at -20°C.

### 2.2 Bone marrow derived macrophage isolation

Bone marrow was isolated from the hind legs of 6–10 week old 129S6/SvEvTac mice (Taconic Biosciences) and filtered through a 100μm filter. Following ACK lysis, the remaining cells were cultured in 20% L929 CM (made in cRPMI) in petri dishes for 6–7 days until differentiated into macrophages. Cells were washed with PBS and fresh 20% L929 CM was added every 2–3 days.

### 2.3 Macrophage polarization and treatment

For experiments where macrophages were treated with cytokines or Wnt3a, BMDMs that had been cultured for at least 6 days in 20% L929 CM were dislodged from the petri dishes by adding 5mM EDTA in PBS and incubating on ice. The reaction was quenched with cRPMI and cells were resuspended in 20% L929 CM at a concentration of 150,000 cells/mL. “M1” macrophages were treated with 50 ng/mL recombinant murine IFN-γ (Peprotech, cat. 315-05) plus 25 ng/mL LPS (Lipopolysaccharide from *E. coli*, Sigma L2630) for the specified amount of time. These doses were previously found to be sufficient to induce production of pro-inflammatory marker genes (20–22). “M2” macrophages were treated with 25 ng/mL recombinant murine IL-4 (Peprotech, cat. 214-14), and Wnt3a-treated macrophages were given 50 ng/mL recombinant Wnt3a (Time Bioscience, cat. rmW3aL-010).

### 2.4 RNA isolation, cDNA synthesis, and RT-qPCR

BMDM RNA was isolated using the RNeasy kit (Qiagen, cat. 74104) with on-column DNase digestion. The iScript cDNA synthesis kit (BioRad, cat. 1708891) was used to make cDNA, which was then diluted 1:16 in nuclease free water before RT-qPCR. RT-qPCR was performed as described in Taylor, et al. (23) and utilizing iTAQ Universal SYBR Green Supermix (BioRad, cat. 1725124) in a 96-well format in the LightCycler 96 (Roche). Differential gene expression analysis was performed following the protocol described in Taylor, et al. (23). All experiments were performed in biologic triplicate with three technical replicates on each plate. All primer pairs (**Supplementary Table 1**) were designed using the NCBI Primer-BLAST (https://www.ncbi.nlm.nih.gov/tools/primer-blast/) tool and validated as described in Taylor, et al. (23). Primers for TATA-box binding protein (TBP) and eukaryotic translation elongation factor 1 alpha 1 (EEF1A1) were both utilized as housekeeping genes in each experiment.

### 2.5 Bulk RNA sequencing

BMDMs were isolated and differentiated as above and then either treated for 5 days with 50 ng/mL Wnt3a or cultured in control 20% L929 CM. Media was changed and fresh Wnt3a added every other day, for a total of 3 treatments in 5 days. On the 5^th^ day, RNA was isolated as above. RNA quality control analysis was performed with Agilent TapeStation, and all samples were determined to have RNA integrity number equivalent (RINe) of 10/10 (highest quality). mRNA enrichment and cDNA library preparation was performed utilizing the the NEBNext^®^ Poly(A) selection kit, and library quality control analysis was performed using Qubit and the Agilent BioAnalyzer. Sequencing was performed at Paired-End 150 bp on the Illumina NovaSeq 6000 targeting an average of 50M reads per sample. Sequencing was performed by the VUMC VANTAGE (Vanderbilt Technologies for Advanced Technologies) shared resource.

### 2.6 Bulk RNA sequencing analysis

Reads were trimmed to remove adapter sequences using Cutadapt (v4.8). Quality control on both raw reads and adaptor-trimmed reads was performed using FastQC (v0.12.1) (www.bioinformatics.babraham.ac.uk/projects/fastqc). Reads were aligned to the Gencode GRCm38.p6 genome using STAR (v2.7.11a). Gencode vM25 gene annotations were provided to STAR to improve the accuracy of mapping. featureCounts (v2.0.6) was used to count the number of mapped reads to each gene. Only protein coding genes were used in the down-stream analysis. ComplexHeatmap was used for cluster analysis and visualization. Significantly differential expressed genes with absolute fold change >= 2 and FDR adjusted p value <= 0.05 were detected by DESeq2 (v1.30.1). For each differential expression comparison, low expressed genes with less than 5 median read count in both conditions were excluded. Genome Ontology and KEGG pathway over-representation analysis was performed on differentially expressed genes using the WebGestalt R package (v1.0.0). Gene set enrichment analysis was performed using GSEA package (v4.3.2) on database (v2024.1.Hs).

### 2.7 Brightfield microscopy and quantification of cell morphology

Live, brightfield images were taken of untreated control BMDMs (M0) and BMDMs treated with Wnt3a (50 ng/mL) for 5 days at 40x magnification on a Leica DMi1 inverted microscope. Image J was used to define cell boundaries and to measure cell area and elongation factor (length of major axis divided by the length of the minor axis for each cell). 3 biologic replicates and a total of 6 fields of view were analyzed for each condition.

### 2.8 Immunofluorescence

IF was performed on BMDMs plated at a concentration of 10,000-30,000 cells per well in glass Nunc Lab-Tek Chamber Slides (Thermo Scientific, 12-565-18). Following treatment with appropriate cytokines or Wnt3a as above, cells were fixed with 3.7% Formaldehyde, permeabilized with 0.5% Triton-X in TBS, and blocked in an antibody dilution buffer (AbDil – 0.1% Triton-X and 2% BSA in TBS) for 30 minutes at room temperature. Cells were stained overnight at 4°C with Anti-MARCO antibody [EPR22944-64] (Abcam, ab239369) or beta-Catenin Rabbit Monoclonal Antibody [D10A8] (Cell Signaling Technology, 8480) diluted 1:100 in AbDil. Cells were then washed and incubated for 1 hour at room temperature with Donkey Anti-Rabbit IgG H&L (Alexa Fluor^®^ 555) (Abcam, ab150074) or Anti-Rabbit IgG H&L (Alexa Fluor^®^ 647) (Invitrogen, A-21245) diluted 1:500 and Hoescht nuclear stain diluted 1:1000 in AbDil. Images were taken on the Nikon Spinning Disk confocal microscope using the 405nm and 561nm (or 640nm) lasers at 4x, 20x, and 60x magnification. Image J was used to quantify mean fluorescence intensity in the 561nm channel (MARCO) in each field of view across 3 biologic replicates.

### 2.9 Western Blot

BMDMs were prepared as above and treated with 50 ng/ml recombinant Wnt3a for 4 hours. Protein collection and cytoplasmic/nuclear fractionation were completed following methods adapted from Thorne, et al. (24). Proteins were resolved by SDS-PAGE and visualized by immunoblotting. Antibodies used were Mouse anti-beta-catenin (1:1000) from Vanderbilt Antibody and Protein Research Core, Mouse anti-GAPDH (1:1000, D4C6R) from CST, and IRDye 800CW Goat anti-Mouse IgG (H+L) (1:10,000, 926-32210) from LICOR.

### 2.10 Flow cytometry of intracellular cytokines

BMDMs were treated with the appropriate cytokines or Wnt3a for the specified amount of time as above. Prior to staining, cells were treated for 4 hours with 1X Brefeldin A solution (BioLegend, 420601) in 20% L929 CM. Cells were then detached from their plates using 5mM EDTA in PBS and resuspended in FACS buffer (2% FBS in PBS). Cells were incubated in BD Pharmingen™ Purified Rat Anti-Mouse CD16/CD32 (BD Biosciences, BDB55314) for 10 minutes at room temperature and stained with 1:4000 Ghost Dye™ Red 780 Viability Dye (Cell Signaling Technology, 18452S) and 1:800 PE/Cyanine7 anti-mouse F4/80 Antibody [BM8] (BioLegend, 123114) for 15 minutes at room temperature in the dark. After surface staining, cells were fixed with Fixation buffer (BioLegend, 420801) for 20 minutes in the dark and resuspended in 1X Intracellular Staining Permeabilization Wash Buffer (BioLegend, 421002). Cells were then stained with APC anti-mouse IL-6 Antibody [MP5-20F3] (BioLegend, 504507), FITC anti-mouse TNF-α Antibody [MP6-XT22] (Biolegend, 506303), and eFluor™ 450 Arginase 1 Monoclonal Antibody [A1exF5] (eBioscience, 48-3697-82) diluted 1:200 in 1X Intracellular Staining Permeabilization Wash Buffer for 20 minutes at room temperature. Unstained BMDMs as well as single-stained control cells or single-stained UltraComp eBeads™ (Thermo Fisher, 01-3333-42) were used as compensation controls. Flow cytometry experiments were performed in the Vanderbilt Flow Cytometry Shared Resource on the BD LSRFortessa™ Cell Analyzer. Analysis of flow cytometry data was performed with FlowJo. The entire gating strategy for all experiments is provided in **Supplementary Figure 1**. The percentage of F4/80^+^IL-6^+^ cells (relative to total live cells) was used to measure IL-6 production. Similarly, the percentage of F4/80^+^TNF-α^+^ or F4/80^+^Arg1^+^ cells (relative to total live cells) was used to measure TNF-α and Arg1 production, respectively.

### 2.11 IL-10-GFP reporter assay

Bone marrow from *Rag1*^-/-^;*Il10*^GFP^;*Rosa26*^lsl-tdTomato^;*Rorc*^Cre^ (25) mice was harvested from 10-week-old mice and macrophages differentiated for 7 days as above. BMDMs were treated with LPS/IFN-γ or Wnt3a for 48 hours before the addition of 1X Brefeldin A in 20% L929 CM for 8 hours. Cells were detached from the plate as above and stained with Ghost Dye™ Red 780 Viability Dye (Cell Signaling Technology, 18452S) and PE/Cyanine7 anti-mouse F4/80 Antibody [BM8] (BioLegend, 123114). Flow cytometry experiments were performed in the Vanderbilt Flow Cytometry Shared Resource on the BD LSRFortessa™ Cell Analyzer. Analysis of flow cytometry data was performed with FlowJo. The gating strategy for this assay is provided in **Supplementary Figure 2**. The percentage of F4/80^+^GFP^+^ cells (relative to total live cells) was used to measure the production of IL-10 by BMDMs with and without Wnt3a treatment.

### 2.12 Migration assays

600μL serum free RPMI with or without 80 ng/mL recombinant CXCL12 (human – PeproTech, 300-28A; murine – PeproTech, 250-20A) or 50 ng/mL recombinant Wnt3a (Time Bioscience, cat. rmW3aL-010) was added to a 24 well plate. A 5.0μm polycarbonate membrane transwell 6.5mm insert (VWR, 10769-236) was added above the media and allowed to equilibrate in the 37°C, 5% CO_2_ incubator for 30 minutes. THP-1 cells (ATCC, #TIB-202) were cultured in an upright flask in cRPMI in suspension at a concentration between 300,000 and 2 million cells/mL in a 37°C, 5% CO_2_ incubator. Bone marrow aspirate was collected as described above. For transwell migration assays, THP-1 cells were resuspended in serum free RPMI at a concentration of 500,000 cells per 100μL, and bone marrow aspirate was resuspended in serum free RPMI at a concentration of 2 million cells per 100μL. Cell suspensions were added to the 5.0μm polycarbonate membrane in 100μL serum free RPMI. Cells were placed in the incubator and allowed to migrate. After 4 hours, the membranes were carefully removed. The media in the 24 well plate was mixed by gently pipetting, and 100μL was added into 3 wells of a white 96 well plate (Fisher Scientific, 07-200-566). 100μL Cell-Titer Glo (Promega, G9683) was added to cells in the 96 well plate and mixed on a rotating platform at 150-200rpm for 25 minutes. Luminescence was read on a BioTek Synergy H1 microplate reader. All luminescence values from individual experiments were normalized using media plus Cell-Titer Glo only wells. Relative migration was reported as a measure of the luminescence of experimental conditions (CXCL12 or Wnt3a) divided by the mean of the luminescence of control wells.

### 2.13 Statistical analysis

**Figure 1:**
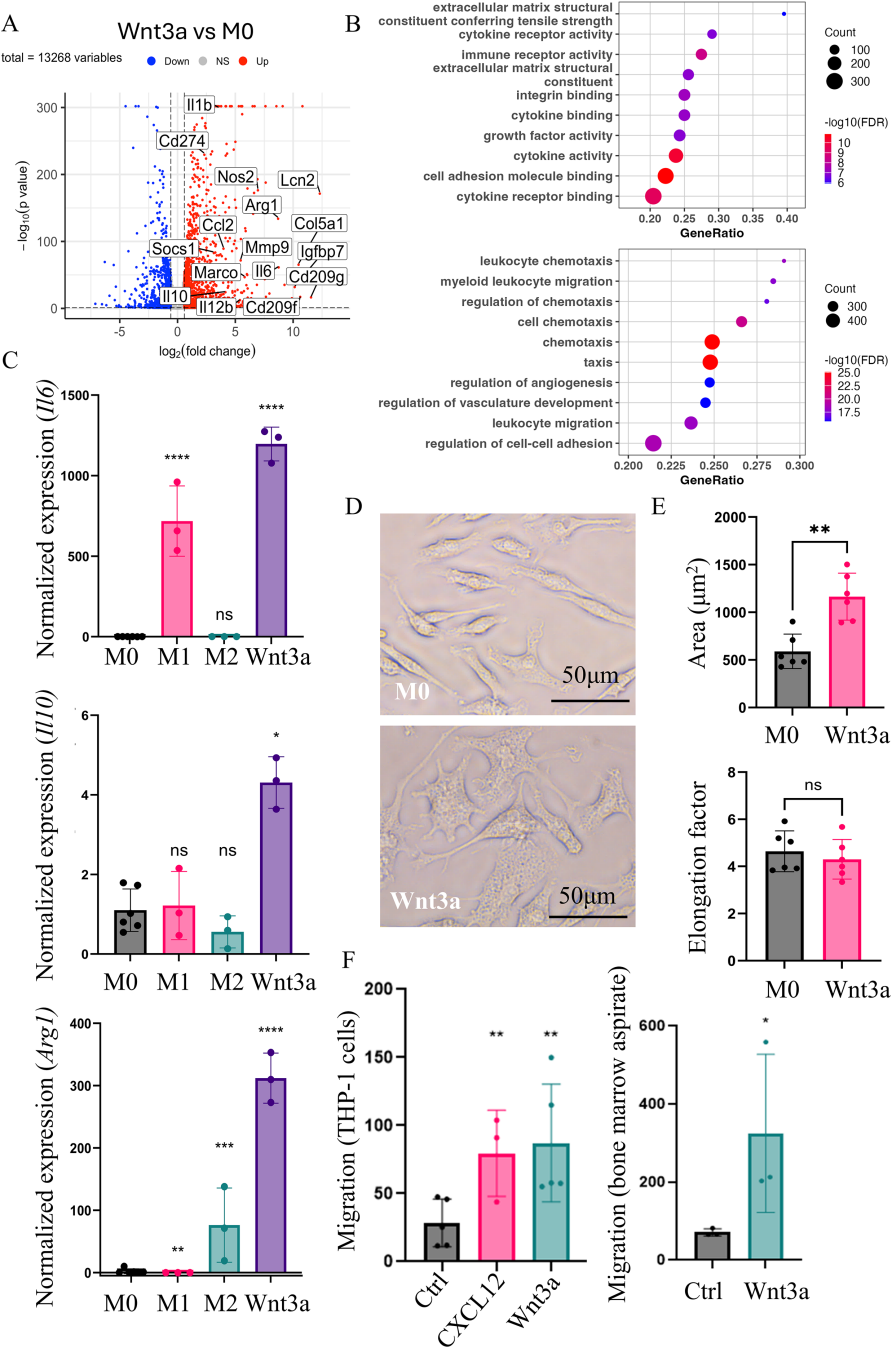
Prolonged exposure to Wnt3a promotes chemotactic and cytokine producing signatures in macrophages. **(A)** A volcano plot highlighting differential gene expression from bulk RNA sequencing of BMDMs treated with 50 ng/mL Wnt3a every other day for 5 days compared to untreated control (M0) BMDMs (n=3). **(B)** Dot plot of top 10 enriched gene sets from Gene Ontology (GO) enrichment analysis (Biological Process and Molecular Function) for differentially expressed genes. **(C)** RT-qPCR analysis of *Il6, Arg1*, and *Il10* gene expression in BMDMs treated with either 25 ng/mL LPS and 50 ng/mL IFN-γ (M1) or 25 ng/mL IL-4 (M2) for 24 hours or 50 ng/mL Wnt3a for 5 days compared to untreated BMDMs (M0) (n=3). **(D)** Representative brightfield images of untreated control (M0) and Wnt3a treated BMDMs. **(E)** Quantification of total cell area and elongation factor, measured by dividing the length of the major axis of each cell by the length of its minor axis, in M0 and Wnt3a treated macrophages (n=3 biologic replicates, 2 fields of view from each replicate were used for quantification). **(F)** Measure of migration of human THP-1 and murine bone marrow aspirate cells across a 5μm transwell membrane towards CXCL12 (80 ng/mL) or Wnt3a (50 ng/mL) in serum free media (n=3 biologic replicates for each experiment). Number of migrated cells were estimated using Cell-Titer Glo^®^ Luminescent Cell Viability Assay. One-way ANOVA was performed on Log2 values for RT-qPCR analysis. *>0.05, **>0.01, ***> 0.001, ****>0.0001. ns, not significant.

**Figure 2:**
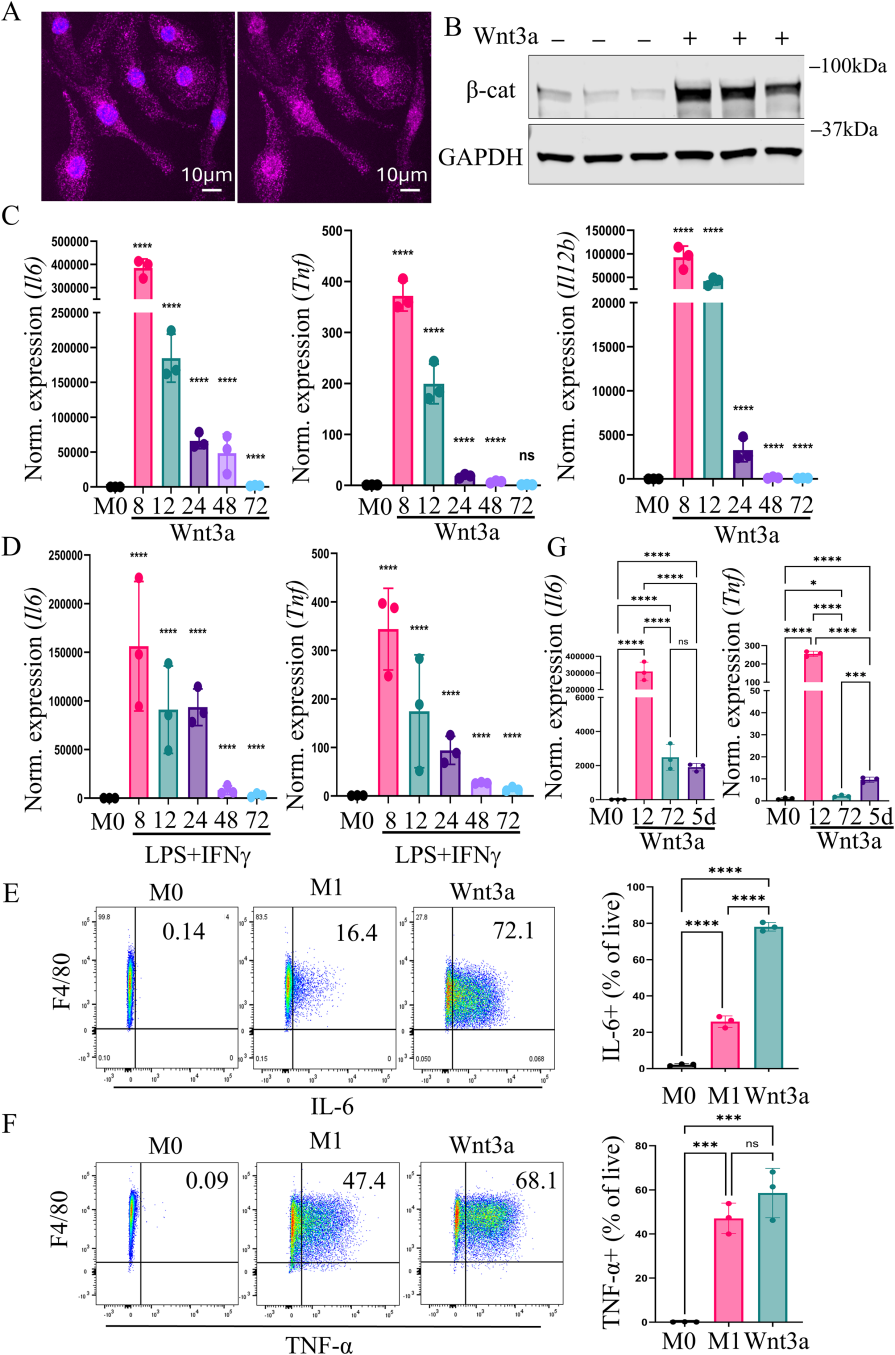
Acutely, Wnt3a treatment causes production of large amounts of inflammatory cytokines, similar to classical macrophage activation. **(A)** Representative fluorescent image of Wnt3a-treated BMDMs immunostained for β-catenin (magenta) and Hoechst nuclear stain (blue) in the left panel. β-catenin staining (magenta) alone is shown in the right panel. **(B)** Western blot of β-catenin and GAPDH in untreated and Wnt3a-treated BMDMs (n=3). BMDMs were treated for 4 hours prior to lysate collection. **(C)** RT-qPCR analysis of the inflammatory cytokines *Il6, Tnf*, and *Il12b* in BMDMs treated with a single dose of 50 ng/mL Wnt3a (n=3 replicates per time point). RNA was isolated after the indicated time points. **(D)** RT-qPCR analysis of the inflammatory cytokines *Il6* and *Tnf* in BMDMs treated with 25 ng/mL LPS and 50 ng/mL IFN-γ over the same time course (n=3 replicates per time point). Representative flow cytometric analysis of **(E)** intracellular IL-6 and **(F)** TNF-α after 8 hours of Wnt3a or cytokine treatment and 4 hours of treatment with 1X Brefeldin to stop secretion of cytokines. Bar graphs show quantification of the percentage of live cells that were **(E)** IL-6 or **(F)** TNF-α positive in 3 biologic replicates. **(G)** RT-qPCR analysis of *Il6* and *Tnf* in BMDMs treated for the indicated amounts of time with 50 ng/mL Wnt3a (n=3 replicates per time point). In 5 day treatments, Wnt3a was replenished every other day and RNA was isolated 24 hours after the last treatment. One-way ANOVA was performed on Log2 values for RT-qPCR analysis. *>0.05, ***> 0.001, ****>0.0001. ns, not significant.

**Figure 3:**
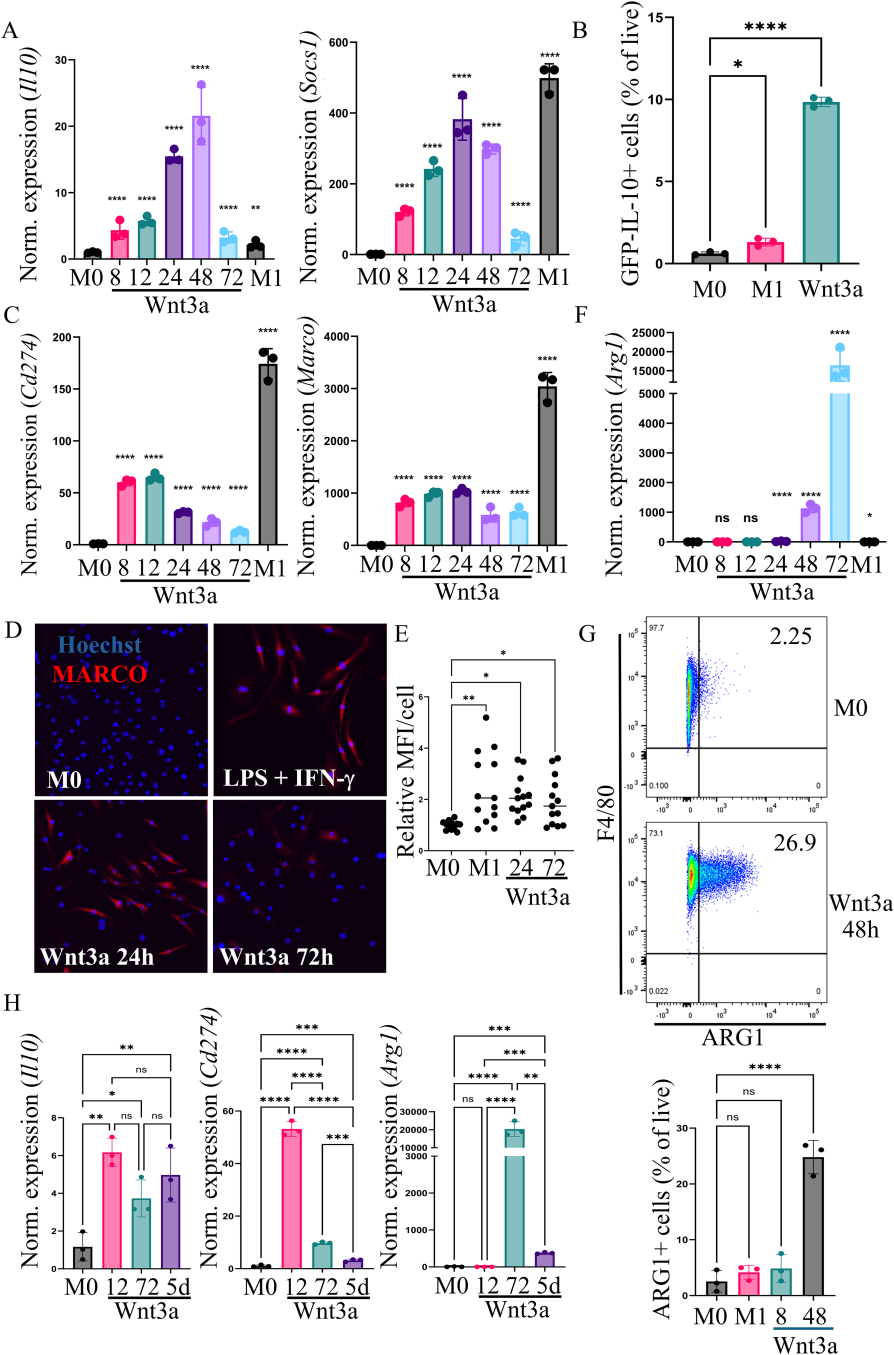
Wnt3a treatment of BMDMs leads to dynamic changes in regulatory and tolerance-associated genes. Where indicated, BMDMs were treated with a single dose of 50 ng/mL Wnt3a for the indicated amount of time or with 25 ng/mL LPS and 50 ng/mL IFN-γ for 24 hours (M1). **(A)** RT-qPCR analysis of negative regulatory genes*, Il10* and *Socs1* (n=3 replicates per time point). **(B)** IL-10 protein expression as indicated by F4/80^+^GFP^+^ cells. Bone marrow derived macrophages from IL-10-GFP reporter mice (*Rag1*^-/-^;*Il10*^GFP^;*Rosa26*^lsl-tdTomato^;*Rorc*^Cre^) were treated with LPS/IFN-γ or Wnt3a for 48 hours and 1X Brefeldin for 8 hours before flow cytometric analysis (n=3). **(C)** RT-qPCR analysis of *Marco* and *Cd274* (n=3 replicates per time point). **(D)** Representative immunofluorescence images of MARCO (red) expression in BMDMs treated with LPS and IFN-γ (M1) or Wnt3a. **(E)** Quantification of MARCO expression by measuring the mean fluorescence intensity per cell in each field of view at 20X magnification in each of the 4 conditions. Each dot represents a different field of view. Three biologic replicates were performed and included in the analysis. **(F)** RT-qPCR analysis of *Arg1* (n=3 replicates per time point). **(G)** Representative flow cytometric analysis of ARG1 after 48 hours of Wnt3a treatment and quantification of the percent of live cells that were F4/80 and ARG1 positive for the indicated treatment conditions (n=3 biologic replicates) **(H)** RT-qPCR analysis of *Il10, Cd274*, and *Arg1* in BMDMs treated for the indicated amount of time with 50 ng/mL Wnt3a (n=3 replicates per time point). In 5 day treatments, Wnt3a was replenished every other day and RNA was isolated 24 hours after the last treatment. One-way ANOVA was performed on Log2 values for RT-qPCR analysis. *>0.05, **>0.01, ***> 0.001, ****>0.0001. ns, not significant.

**Figure 4:**
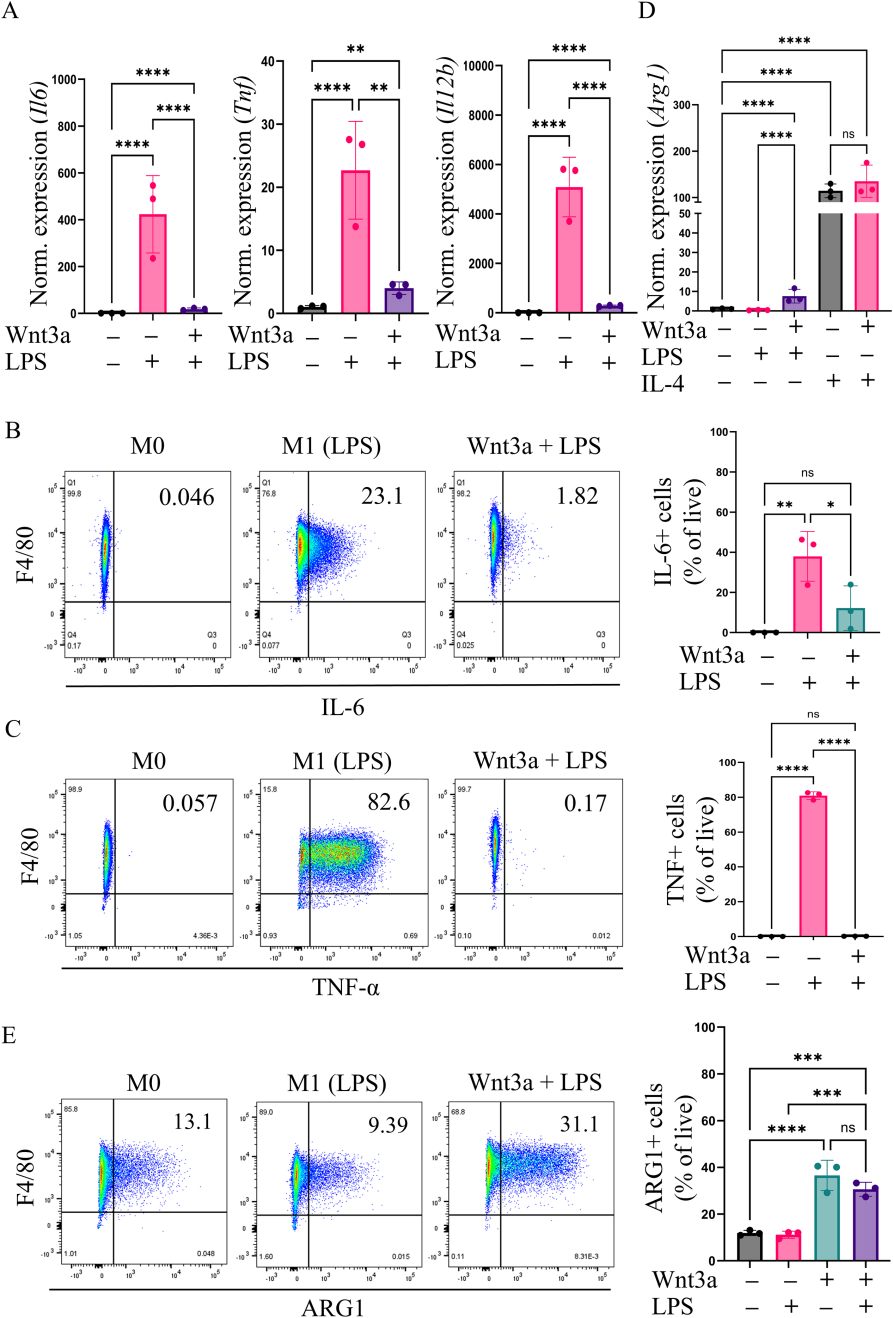
Prolonged Wnt3a treatment leads to macrophage tolerance and a decreased ability to produce inflammatory cytokines. Where indicated, Wnt3a stimulation consisted of 3 treatments of 50 ng/mL Wnt3a over 5 days before adding additional cytokines. LPS (25 ng/mL) or IL-4 (25 ng/mL) were given for 8 hours where indicated. **(A)** RT-qPCR analysis of the inflammatory cytokines *Il6, Tnf*, and *Il12b* (n=3). **(B)** Flow cytometric analysis of intracellular IL-6 levels after 4 hours of treatment with 1X Brefeldin to stop secretion of cytokines. Bar graph shows quantification of the percentage of live cells that were IL-6+ in 3 biologic replicates **(C)** Flow cytometric analysis of intracellular TNF-α levels after 4 hours of treatment with 1X Brefeldin. Bar graph shows quantification of the percentage of live cells that were TNF-α+ in 3 biologic replicates. **(D)** RT-qPCR analysis of the “anti-inflammatory” cytokine *Arg1* (n=3). **(E)** Flow cytometric analysis of intracellular ARG1 levels after 4 hours of treatment with 1X Brefeldin. Bar graph shows quantification of the percentage of live cells that were ARG1+ in 3 biologic replicates. One-way ANOVA was performed on Log2 values for RT-qPCR analysis. *>0.05, **>0.01, ***> 0.001, ****>0.0001. ns, not significant.

All experiments were performed with at least 3 biological and 3 technical replicates unless otherwise noted. Data are represented as mean +/- standard deviation. For RT-qPCR experiments, data on log2 scale was compared by one-way ANOVA with a *post-hoc* test: either Dunnett’s test for comparisons of multiple treatment groups to the control group or Tukey’s test for pairwise comparisons (**Figure 1C**, **2C, D, G**, **3A, C, F, H**, **4A, D**, **Supplementary Figure 3**, **Supplementary Figure 4**, and **Supplementary Figure 5**). For total cell area and elongation factor measurements, Welch’s t-test was used to compare two independent groups (**Figure 1E**). For migration of human THP-1 and murine bone marrow aspirate cells, the number of migrated cells was analyzed on a natural log scale using a mixed-effect model. This model assesses the association between treatment and outcome, accounting for correlated observations within replicates. Least-squares means (model-based estimates) were used to summarize outcomes by group, and group differences were evaluated using a Wald test. *Post-hoc* comparisons were adjusted for multiple testing using Tukey’s method (**Figure 1F**). For immunofluorescence images of MARCO expression, mean fluorescence intensity per cell in each field of view (n=12, across 3 biological replicates) was compared across the four conditions using one-way ANOVA with *post-hoc* testing (**Figure 3E**). For flow cytometric analyses, the percentage of live cells expressing the measured marker/cytokine was compared among groups using one-way ANOVA with *post-hoc* tests (**Figures 2E, F**, **3B, G**, **4B, C, E**, and **Supplementary Figure 4C**). All statistical analyses were performed using GraphPad Prism software or R version 4.5.1. We consider *P value*s < 0.05 as significant. *, p < 0.05; **, p < 0.01; ***, p < 0.001; and **** p < 0.0001.

## 3 Results

### 3.1 Long-term Wnt3a treatment supports the expression of pro- and anti-inflammatory cytokines in macrophages and acts as a monocyte chemoattractant

Macrophages in a Wnt-ligand rich tissue microenvironment continuously experience Wnt pathway activation. To mimic this environment *in vitro*, we treated BMDMs every other day for 5 days with 50 ng/mL Wnt3a. We then performed next generation sequencing on these Wnt-stimulated and untreated (M0) BMDMs to analyze their gene expression. Differential expression analysis demonstrated induction of pro-inflammatory cytokines, such as *Il6* and *Il1b*, and anti-inflammatory or regulatory factors, including *Socs1* and *Il10*, with Wnt3a treatment (**Figure 1A**; **Supplementary Table 2**). Additionally, both *Nos2* and *Arg1*, which compete for the substrate L-arginine and have opposing effects on immune regulation (26), were increased in Wnt3a-treated macrophages compared to untreated controls. Functional enrichment analysis of biologic process and molecular function gene ontologies revealed upregulation of genes involved in chemotactic, cytokine, and growth factor-related functions (**Figure 1B**).

To confirm the RNA sequencing results, we performed RT-qPCR analysis of select upregulated genes. We compared the expression of these genes in Wnt3a-treated BMDMs, classically activated (“M1”, stimulated with LPS and IFN-γ for 24 hours), and alternatively activated (“M2”, stimulated with IL-4 for 24 hours) macrophages. Both pro-inflammatory genes that were highly increased in M1-like BMDMs (e.g. *Il6*) and anti-inflammatory genes that were increased in M2-like BMDMs (e.g. *Arg1*) were significantly expressed in Wnt3a-treated BMDMs (**Figure 1C**). Other genes that were not specific to either M1- or M2-like macrophages (e.g. *Il10*) were also confirmed to be upregulated in Wnt3a-treated BMDMs. Additionally, Wnt3a-treated BMDMs appeared to have a distinct morphology. Brightfield microscopy revealed flattening and increased projections after 5 days of Wnt3a treatment (**Figure 1D**). Further analysis demonstrated that these macrophages had significantly increased cell areas, as has been seen in macrophages treated with LPS and IFN-γ (27) (**Figure 1E**). However, there was no difference in their degree of elongation, which has been shown to be increased in alternatively-activated macrophages (28).

Given that Wnt ligands and Wnt signaling are known to be important in regulating cell migration in development and in some cancers (29–31), we next assessed the ability of Wnt ligands to act as monocyte chemoattractants. We found that recombinant Wnt3a could directly attract both THP-1 cells and murine bone marrow aspirate cells across a transwell membrane in a manner similar to the known chemoattractant, CXCL12 (**Figure 1F**). This effect supported the gene ontology results and built upon a previous report that Wnt3a-treated macrophage conditioned media could enhance monocyte migration (32).

### 3.2 Wnt3a induces a rapid burst of inflammatory cytokine production in macrophages

Recombinant Wnt3a activates Wnt/β-catenin signaling in BMDMs, as evidenced by nuclear localization of β-catenin and increased β-catenin expression (**Figures 2A, B**). To study acute and persistent gene expression changes in macrophages following Wnt activation, we collected RNA at 8, 12, 24, 48, and 72 hours after treating BMDMs with a single dose of recombinant Wnt3a. We then performed RT-qPCR to evaluate gene expression in Wnt-treated macrophages over time. In contrast to most published studies, Wnt3a-treated BMDMs had profound expression of the pro-inflammatory cytokines *Il6, Tnf*, and *Il12b* at early time points (**Figure 2C**). This mirrored the expression of these cytokines in BMDMs treated with LPS and IFN-γ (“M1” macrophages) over the same time course (**Figure 2D**). We also evaluated protein-level expression of IL-6 and TNF-α using flow cytometry and confirmed that Wnt3a-treated macrophages and classically activated macrophages both produced robust IL-6 and TNF-α 8 hours after treatment (**Figures 2E, F**). Finally, we saw that after prolonged exposure to Wnt3a (5 days), inflammatory cytokine production was drastically decreased compared to acute time points, though levels remained higher than unstimulated macrophages (**Figure 2G**). This finding of an acute inflammatory phenotype in Wnt3a-treated macrophages challenges the current paradigm that Wnt signaling promotes M2-like macrophage polarization (14, 15).

### 3.3 Dynamic gene expression changes in Wnt3a-treated macrophages mimic classically activated macrophages

Given that inflammatory cytokine production decreased in both Wnt3a-stimulated and classically activated (“M1”) macrophages over time (**Figures 2C, D**), we next chose to examine genes known to be involved in feedback inhibition of the inflammatory response: *Socs1* and *Il10*, which inhibit inflammatory signaling cascades and actively repress transcription of inflammatory genes, respectively (33). Expression of both *Il10* and *Socs1* peaked 24–48 hours after stimulation with Wnt3a (**Figure 3A**), which was well after the initial increase in inflammatory cytokine production. BMDMs from IL-10-GFP reporter mice (25) were used to validate the production of IL-10 following 48 hours of treatment with Wnt3a. The percentage of live cells that were F4/80 and GFP positive was significantly higher in Wnt3a-treated compared to control BMDMs (**Figure 3B** and **Supplementary Figure 2**) The patterns of expression of *Il10* and *Socs1* were mirrored in “M1” BMDMs (**Supplementary Figure 3A**). Alternatively-activated (“M2”) macrophages, on the other hand, showed little expression of *Il10*, and their expression of *Socs1* was highest at early, rather than late, time points (**Supplementary Figure 3B**). To further characterize the dynamic response of macrophages to Wnt3a-treatment, we evaluated the expression of *Cd274*, the gene that encodes PD-L1, which has well recognized roles in inhibiting the adaptive immune response but has also been shown to regulate inflammatory macrophage phenotypes (34, 35), and *Marco*, a scavenger receptor that is increased in tolerant macrophages (36, 37). Again, the expression patterns in Wnt3a-treated BMDMs and “M1” BMDMs were similar for these two genes (**Figures 3C–E** and **Supplementary Figure 3A**). “M2” macrophages expressed much lower levels of *Cd274* and had less *Marco* expression than even untreated (“M0”) BMDMs (**Supplementary Figure 3B**). This data showing expression patterns of several regulatory genes in “M1”, “M2”, and Wnt3a-treated macrophages, supports the hypothesis that Wnt3a-stimulated macrophages resemble classically activated, “M1” macrophages.

To strengthen this hypothesis, we wanted to examine a gene typically associated with “M2” macrophage polarization. *Arg1* is often associated with alternative macrophage activation but is also expressed late after classical activation (38, 39). Evaluation of *Arg1* gene expression and protein production revealed that both Wnt3a-stimulated BMDMs and “M1” macrophages exhibited high levels of *Arg1*48 hours after initial treatment (**Figures 3F, G** and **Supplementary Figure 4A**). In contrast, “M2” macrophages began expressing *Arg1* by 8 hours post IL-4 stimulation (**Supplementary Figures 4B, C**). Finally, RT-qPCR comparing gene expression of acute and prolonged Wnt3a stimulation (5 days) demonstrated a dampened response after continual Wnt activation for most genes, except *Il10*, a known mediator of macrophage tolerization (**Figure 3H**) (33). Our data supports a model where Wnt3a treatment, rather than promoting true “M2” polarization, triggers acute inflammatory activation of macrophages, which then, over time, transition to a tolerant or “anti-inflammatory”-like phenotype.

### 3.4 Wnt3a induces a tolerant phenotype of macrophages

To directly test the ability of Wnt3a to tolerize macrophages, we added inflammatory or anti-inflammatory stimuli to BMDMs pretreated for 5 days with Wnt3a and measured cytokine expression with RT-qPCR and flow cytometry (**Figures 4A–C**). Pretreatment with Wnt3a greatly reduced the capacity of these macrophages to express pro-inflammatory cytokines *Il6, Tnf*, and *Il12b* in response to LPS-stimulation. Additionally, IFN-γ pretreatment, which has been shown to rescue toll-like receptor (TLR)-induced tolerance (40, 41), partially restored the expression of some inflammatory cytokines in Wnt3a-induced tolerance (**Supplementary Figure 5**). We did not observe any significant additive effect of Wnt3a pretreatment on IL-4 stimulated expression of the anti-inflammatory cytokine *Arg1* (**Figure 4D**). However, *Arg1* expression was increased after LPS stimulation in Wnt3a-treated BMDMs compared to control BMDMs (**Figures 4D, E**). These results support a role for chronic Wnt3a treatment in promoting a tolerant phenotype of macrophages, as they fail to robustly respond to inflammatory stimuli but maintain the ability to express the anti-inflammatory gene *Arg1*.

## 4 Discussion

Inhibition of the Wnt pathway is being investigated as a therapeutic strategy in cancer and other diseases of chronic inflammation where macrophages play important roles in the tissue microenvironment; however, past research has supported contradictory conclusions about the effects of Wnt treatment on macrophage polarization. Our evaluation of Wnt stimulation in isolation of other stimuli, over a well-defined time course, found that Wnt3a-treated BMDMs acutely produced abundant inflammatory cytokines. Shortly after this initial inflammatory phase, Wnt3a-treated macrophages began to express regulatory genes, such as *Il10* and *Socs1*. *Arg1*, a gene that rapidly rises in IL-4-treated macrophages, did not peak in Wnt3a-stimulated macrophages until more than 48 hours after treatment. These dynamic changes in gene expression closely resembled an LPS-induced inflammatory response. To mimic the chronic exposure of macrophages to Wnt ligands that could be encountered in a Wnt-ligand-rich tumor microenvironment or injury site, we stimulated macrophages *in vitro* with Wnt3a continuously for 5 days. Gene expression analysis revealed that treated macrophages had increased chemotactic signatures and cytokine production. Further comparison of these macrophages to macrophages acutely stimulated with Wnt3a showed an overall decrease in both inflammatory and anti-inflammatory cytokine production with chronic Wnt3a treatment. We then tested the ability of our Wnt3a-exposed BMDMs to respond to LPS stimulation. We found that they had a greatly diminished ability to produce inflammatory cytokines, suggesting Wnt3a exposure could directly tolerize macrophages.

Despite having some common phenotypic markers, tolerant macrophages do differ from M2-like macrophages in their origin and likely in their functional roles (33, 39, 40). Classically, macrophage tolerance occurs after initial exposure to TLR ligands, such as LPS (40). After an acute inflammatory phase, crucial regulatory mechanisms shift these macrophages towards a refractory state, and subsequent exposure to TLR ligands induces a dramatically dampened response (40, 42). Plasticity is a key aspect of macrophage biology, and the ability to undergo this shift is important for immune regulation. Based on our data, we hypothesize that Wnt ligands recruit monocytes into a tissue/tumor and initiate a brief inflammatory response that is controlled by intrinsic negative feedback mechanisms. Prolonged environmental exposure to Wnt ligands would then cause macrophages to become tolerant to additional inflammatory stimuli and to adopt a phenotype resembling, but distinct from, alternatively activated macrophages. Our results are supported by previous work demonstrating that some Wnt ligands can act directly as TLR2/4 ligands (43). Overall, our results help to reconcile the discordant observations that Wnt/β-catenin signaling can promote M1- or M2-like macrophage polarization (14–18) and provide further insight into the direct effects of Wnt stimulation on macrophage phenotype.

The current study, while important, does have several limitations that should be considered in future research. While we employed an *in vitro* system to focus on the direct effects of Wnt3a, we acknowledge that the *in vivo* tissue microenvironment is far more complex, with diverse contextual signals that could modulate macrophage behavior. We also recognize that *in vivo* macrophages are both plastic and heterogeneous. The conventional classification of macrophages as “M1-like” or “M2-like” is an oversimplification that fails to capture the full phenotypic spectrum, a limitation underscored by our findings. Macrophages treated with Wnt3a likely display a distinct phenotype that does not fit neatly within this model yet still contributes to macrophage functionality. Finally, this study was focused on better understanding the effects of Wnt pathway activation on macrophages and did not directly test the effects of broad Wnt pathway inhibition on macrophage phenotype in either an *in vitro* or *in vivo* system, which would be important for future studies to consider.

Upregulation of Wnt signaling in the tumor microenvironment is common and often predicts T cell and antigen presenting dendritic cell exclusion (7, 8). Because of this, Wnt/β-catenin signaling is already being investigated as a potential therapeutic option for cancer, especially in conjunction with immune checkpoint inhibitors (9, 11, 44). Having a thorough understanding of how Wnt signaling affects all cell types within a stromal microenvironment is crucial for predicting how different tumors may respond to these inhibitors. Previous literature suggests that Wnt signaling could increase the expression of M2-like marker genes in response to IL-4 stimulation and create macrophages that support tumor growth (14–16). However, a few studies contradict this paradigm and postulate that, in fact, Wnt signaling promotes inflammatory phenotypes of macrophages in some settings (17, 18). One reason for this apparent contradiction is that this research was conducted in different contexts with various additional stimuli and over different time periods, an important distinction given the natural plasticity of macrophages. Our study shows that Wnt3a stimulation alone causes dynamic changes in cytokine production and regulatory gene expression that closely resemble an “M1” macrophage expression profile, thus highlighting the importance of studying these effects on macrophages in isolation and over different time courses.

Given the broad interest in Wnt/β-catenin signaling as a therapeutic target in cancer and other diseases of chronic inflammation, this study provides important insight into the role of Wnt activation in macrophage biology. It expands upon previous research by demonstrating that Wnt signaling produces time-dependent effects on macrophage phenotype. Additionally, it supports a novel role for Wnt3a as both an activator of inflammatory signaling and an inducer of macrophage tolerance.

## Data Availability

The datasets presented in this study can be found in online repositories. The names of the repository/repositories and accession number(s) can be found below: https://www.ncbi.nlm.nih.gov/bioproject/PRJNA1367563, PRJNA1367563.
