# Supplementary material for: Prolonged Wnt3a exposure tolerizes macrophages to inflammatory stimuli
Source: Front Immunol. 2026 Mar 12;17:1752131. doi: 10.3389/fimmu.2026.1752131 (PMC13017329; doi:10.3389/fimmu.2026.1752131)
Supplement: Supplementary file 1 [file DataSheet1.docx]

Supplementary Material

**Supplementary Table 1**. Primers used for RT-qPCR.

| Gene | Forward Primer Sequence | Reverse Primer Sequence |
| --- | --- | --- |
| mouse EEF1A1 | TCCGATTACGACGATGTTGA | AGTCGCCTTGGACGTTCTT |
| mouse TBP | AGCTCTGGAATTGTACCGCA | AATCAACGCAGTTGTCCGTG |
| mouse TNF | GGTGCCTATGTCTCAGCCTC | GCTCCTCCACTTGGTGGTTT |
| mouse IL12b | TTGTTCGAATCCAGCGCAAG | TTCTCTACGAGGAACGCACC |
| mouse IL6 | AGACAAAGCCAGAGTCCTTCA | TCTTGGTCCTTAGCCACTCCT |
| mouse ARG1 | GGTGGATGCTCACACTGACA | TACGTCTCGCAAGCCAATGT |
| mouse MARCO | GCACTGCTGCTGATTCAAGTTC | AGTTGCTCCTGGCTGGTATG |
| mouse CD274 | TGCGGACTACAAGCGAATCACG | CTCAGCTTCTGGATAACCCTCG |
| mouse IL10 | ACAGCCGGGAAGACAATAACT | TAAGGCTTGGCAACCCAAGTA |
| mouse MMP9 | TAGATCATTCCAGCGTGCCG | GCCTTGGGTCAGGCTTAGAG |
| mouse SOCS1 | CTTCCGCTCCCACTCCGATT | CCGAAGCCATCTTCACGCTG |


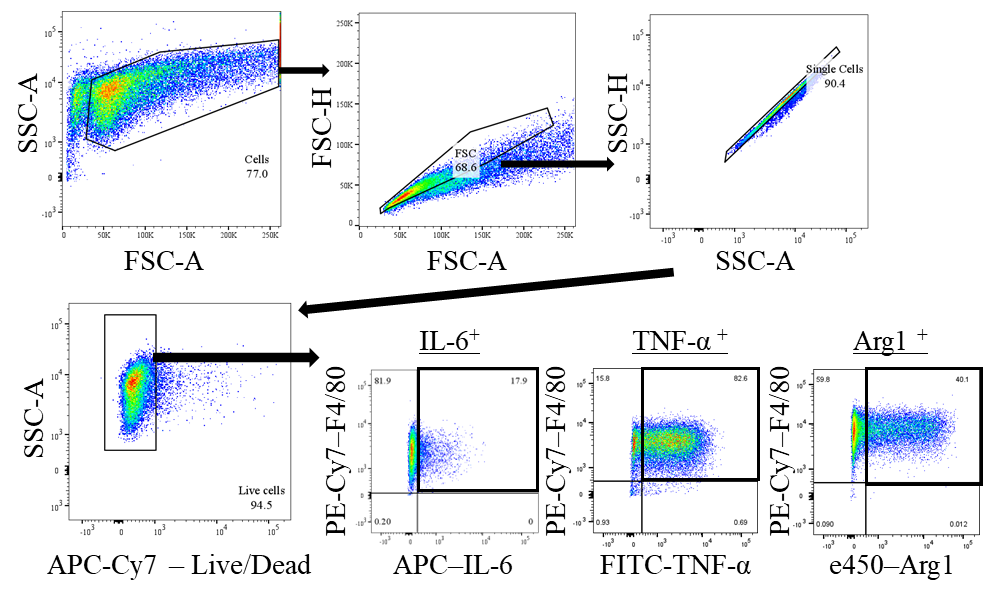
**Supplementary Figure 1.** Representative gating strategy for flow cytometric analysis of intracellular IL-6, TNF-α, or Arginase 1 levels. Cells were gated based on forward and side scatter, doublets were excluded as above, and then live cells (those negative for Ghost Dye™ Red 780 Viability Dye) were gated on their expression of F4/80 and the cytokine/marker of interest. The percentage of live cells that were F4/80^+^ and IL-6/TNF/Arg1^+^ were quantified for each condition.

**
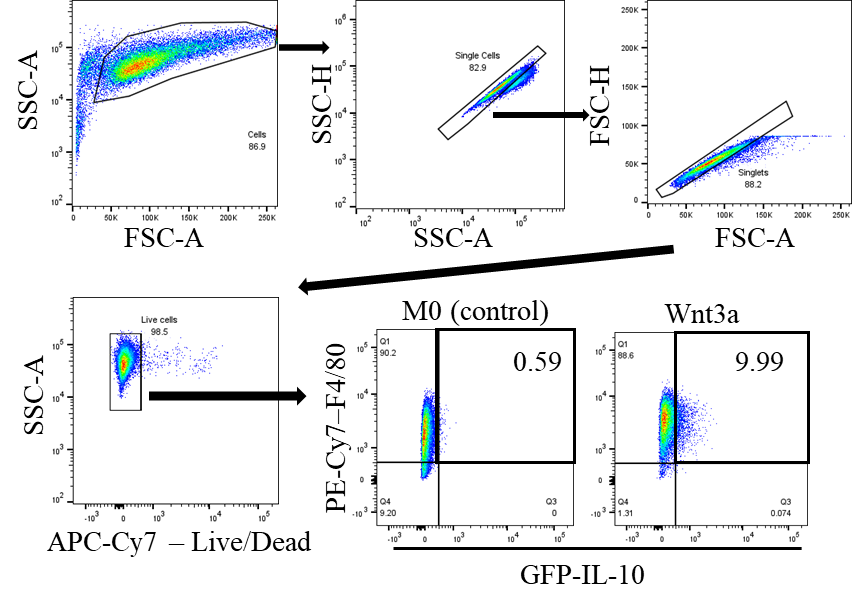
Supplementary Figure 2. G**ating strategy and representative flow cytometric analysis of GFP levels in BMDMs from IL-10-GFP reporter mice. The percentage of live cells that were F4/80^+^ and GFP^+^ were quantified to predict the expression of IL-10.


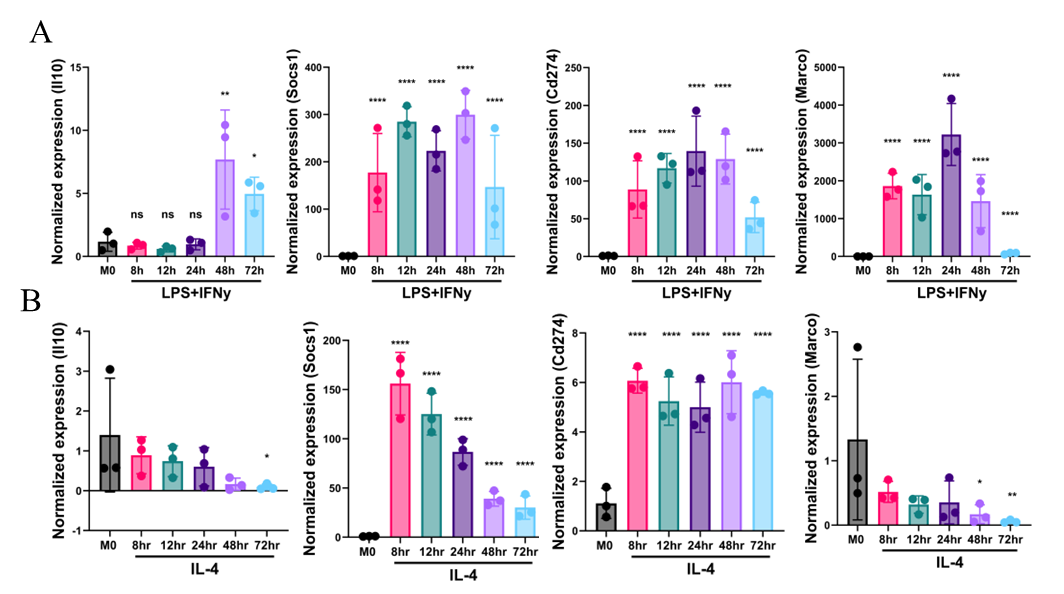


**Supplementary Figure 3.** Time-course gene expression in BMDMs following treatment with LPS and IFN-γ or IL-4 (n=3). **(A-B)** RT-qPCR analysis of regulatory and tolerance-associated markers *Il10, Cd274, Marco,* and *Socs1* in BMDMs treated for the indicated amount of time with **(A)** 25 ng/mL LPS + 50 ng/mL IFN-γ or **(B)** 25 ng/mL IL-4 for the indicated amount of time. One-way ANOVA was performed on Log2 values. * >0.05, **>0.01, *** > 0.001, **** >0.0001.


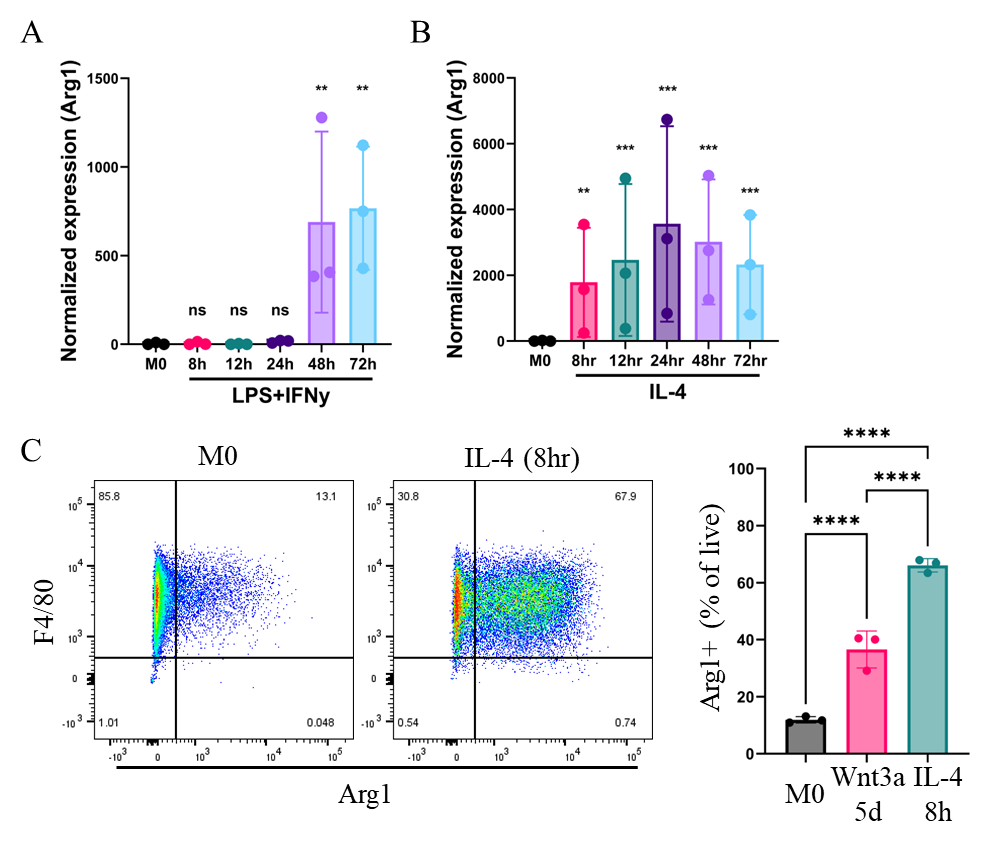


**Supplementary Figure 4.** Expression of *Arg1* in BMDMs following treatment with LPS and IFN-γ or IL-4 (n=3). **(A-B)** RT-qPCR analysis of *Arg1* in BMDMs treated for the indicated amount of time with **(A)** 25 ng/mL LPS and 50 ng/mL IFN-γ or **(B)** 25 ng/mL IL-4 for the indicated amount of time. One-way ANOVA was performed on Log2 values. **(C)** Representative flow cytometric analysis of control (M0) and IL-4 treated macrophages. Bar graph represents F4/80+Arg1+ population (as a percentage of live cells) in control, chronic Wnt3a-treated, and IL-4 treated BMDMs (n=3). * >0.05, **>0.01, *** > 0.001, **** >0.0001.


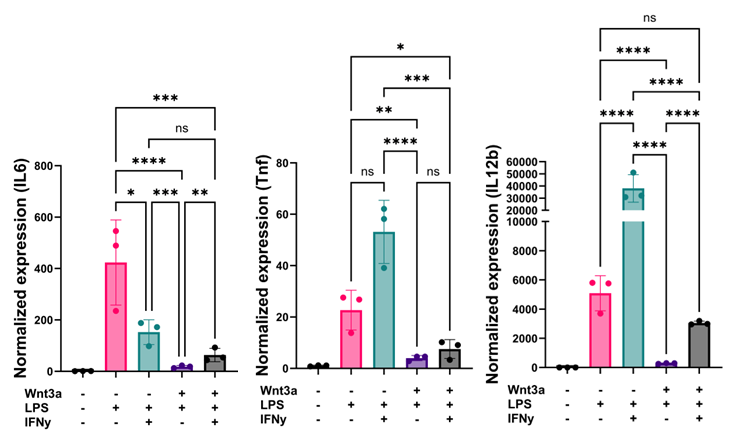


**Supplementary Figure 5.** Extended RT-qPCR data showing Wnt3a-induced tolerance in BMDMs. Where indicated, Wnt3a stimulation consisted of 3 treatments of 50 ng/mL Wnt3a over 5 days before adding additional cytokines. IFN-γ (50 ng/mL*)* was given as a pretreatment 12 hours prior to LPS stimulation (25 ng/mL for 8 hours) where indicated. RT-qPCR analysis of the inflammatory cytokines *Il6, Tnf*, and *Il12b* (n=3).
